# Informal caregiving among people supporting a person with type 2 diabetes in rural communities of Northern Vietnam: A cross-sectional study of caregiver burdens

**DOI:** 10.1371/journal.pone.0304821

**Published:** 2024-05-31

**Authors:** Dieu Huyen Thi Bui, Bai Xuan Nguyen, Jens Søndergaard, Tine M. Gammeltoft, Ib Christian Bygbjerg, Jannie Nielsen, Dan Wolf Meyrowitsch

**Affiliations:** 1 Thai Binh University of Medicine and Pharmacy, Thai Binh, Vietnam; 2 The Research Unit for General Practice, Department of Public Health, University of Southern Denmark, Odense C, Denmark; 3 Department of Anthropology, University of Copenhagen, Copenhagen K, Denmark; 4 Department of Public Health, Global Health Section, University of Copenhagen, Copenhagen K, Denmark; 5 Department of Public Health, Section of Social Medicine, University of Copenhagen, Copenhagen K, Denmark; Jinnah Sindh Medical University, PAKISTAN

## Abstract

**Objective:**

The prevalence of type 2 diabetes mellitus (T2DM) in Vietnam has doubled from 3% to 6% over the last decades, with potential consequences for persons with diabetes and their caregivers. This study aimed to assess caregiver burdens and factors associated with caregiver burden.

**Method:**

A cross-sectional study was conducted in 2019, using data from 1,241 informal caregivers (ICGs). Caregiver burden was scored from 0–32 using 8 questions from the Zarit Burden Interview (ZBI). Quantile regression analysis was used to identify factors associated with caregiver burden.

**Results:**

The median score of the ZBI was 7.0 (Q1-Q3: 2.0–10.0), indicating that the burden among caregiver of persons with T2DM is not high. Quantile regression showed that the higher the monthly income, the lower the burden among caregivers (50% quantile and 75% quantile of burden: -0.004). Lower educational level (25%Q: 4.0, 50%Q; 3.0, 75%Q: 2.16), being a farmer (25%Q: 2.0) and providing care to other people besides the person with T2DM (25%Q: 2.0, 50%Q; 2.54, 75%Q: 1.66) were associated with higher burden on caregivers.

**Conclusion:**

The study found that caregivers facing additional life stressors, such as low income or other caregiving responsibilities, reported higher levels of burden. These findings could inform the development of interventions targeted at supporting informal caregivers in rural areas in low- and middle-income countries.

## Introduction

Type 2 Diabetes mellitus (T2DM) is a chronic non-communicable disease with substantial risk of complications such as cardiovascular disease, kidney failure, blindness and lower limb amputations [1]. T2DM requires daily treatment and management, including a healthy and balanced diet and physical activity, and daily intake of medicine and blood glucose measurements [2, 3] For many persons with T2DM, these tasks are supported by their social relations to family members, friends [4] and neighbours [5]. These support persons are commonly named informal caregivers (ICGs), and they provide many forms of support, such as accompanying the person with T2DM to hospital appointments or helping inject insulin, as well as social and emotional support [6]. In addition to having to spend time taking care of the patient, ICGs often have to pay for the patients’ medicine and medical care [7]. Previous studies have showed that higher levels of caregiver support are associated with better glycemic control, lower mortality and less diabetes-related distress [8]. In addition, compared to participants with no ICG, those with an ICG are more likely to report moderate or high diabetes medication adherence [9]. The role of ICGs also includes assisting in mobility, personal care, performing household duties and solving/addressing financial problems and thus increasing the quality of life of persons with T2DM [10].

The number of tasks of caring, the development of associated complications and higher caregiving needs may contribute to a significant feeling of burden among caregivers, commonly referred to as the caregiver burden [11]. Burden can lead to negative consequences for caregivers and persons with diabetes such as increased risk of depression [12]. Researchers have reported that most family members experience a sense of burden when providing care for persons with diseases, primarily due to challenges associated with long-term home care [13]. Furthermore, for persons with a spouse diagnosed with diabetes, the risk of depression was found to be even higher when diabetes was accompanied by comorbidities [12, 13]. A study conducted in Nigeria found that females were found to be four times more likely to experience a high burden of caregiving [14], while other studies showed that factors affecting the caregivers’ burden were caregivers’ age, personal diabetes status, family structure, caregivers’ education level, caregivers’ income level and hours spent per week for the care of the persons with T2DM [15–17].

A systematic review reported that the pooled prevalence of T2DM in Vietnam has approximately tripled, increasing from 3% in the period 2000 to 2004 to 9% in the period 2016 to 2020 [18]. Primary health care in Vietnam can presently not provide the necessary capacity and technologies needed to help persons with T2DM to properly manage their disease [19], so self-management is highly important [7, 20]. The rapid increase in the number of persons with T2DM in Vietnam is expected to lead to a critical demand for informal caregiving.

Traditional values and family norms in Vietnam reflect a Confucian heritage [21], where it is considered an obligation and responsibility of family members, especially children, and other relatives to look after older persons and sick persons [22]. In Vietnam, nearly 6 million persons with T2DM potentially receive informal care from a family member. Yet, how these family members perceive caregiving and caregiver burdens is not known. Further, it is not known how caregiver burden is associated with gender, age, educational level, income level of the caregiver, relationship between caregiver and the person with T2DM and the duration of caring. Therefore, we conducted a cross-sectional study to assess the perceived caregiver burden among ICGs supporting persons with T2DM in a rural area of Vietnam and to identify the factors associated with caregiver burden.

## Materials and methods

### Study population and setting

**Fig 1:**
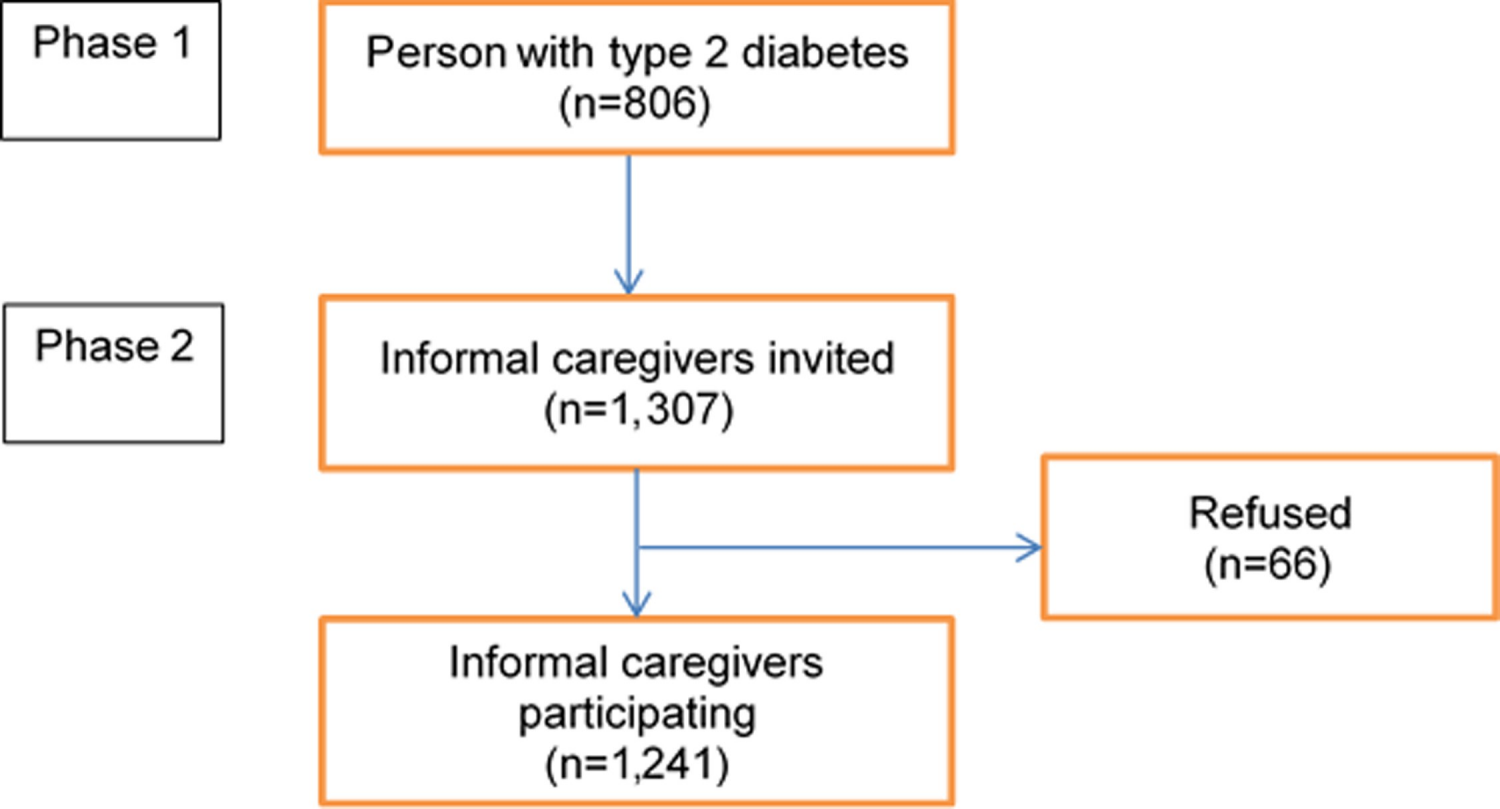
Flowchart of recruitment of informal caregivers.

We conducted a cross-sectional survey from April to July 2019 in Thai Binh Province in northeastern Vietnam. The total population of Thai Binh Province is 1.86 million people, the GDP per capita is 1650 USD, and more than 90% of the population in the province lives in rural areas [17]. Thai Binh Province is subdivided into seven districts. We employed random sampling to select two districts by using the random number function (RAND) in Microsoft Excel, namely Quynh Phu District in the north and Vu Thu District in the south. Within each district, using medical records from district hospitals, we identified the two communes with the highest prevalence of individuals with T2D. A neighboring commune of each of the four identified communes was selected randomly for the sake of convenience in data collection. In this manner, a total of 8 communes were chosen for inclusion in the study. In the selected communes, lists of individuals previously diagnosed with T2DM were obtained from the three district hospitals. In the first phase of the project, all 963 people with T2D were invited directly to participate in the study with assistance from the village health workers; 37 respondents declined to participate, 78 respondents were absent at the time of study, and 42 respondents were excluded because they could not recall their age at the time of diagnosis. These criteria resulted in a cohort of 806 people with T2D (Fig 1). In the second phase of the project, we asked 806 respondents to list their two most important ICGs. In the second phase of the study, only 1,307 ICGs were invited to participate as 305 people only listed one ICG. The inclusion criteria of ICGs were that they had to be 14 years of age or older (100% of ICGs fulfilled this criterion) and willing to participate in the survey. Among those, 66 individuals refused to participate in the study (5.1%). Hence, a total of 1,241 ICGs were included in the study (acceptance rate: 94.9%) (Fig 1).

### Ethical approval

The study was approved by the Medical Ethics Committee of Thai Binh University of Medicine and Pharmacy, Vietnam (decision 11/2018, November 23, 2018). Before each interview, written informed consent was obtained from each participant. Participants could withdraw at any time during the interview. The participants were interviewed in their homes, or another place selected by them. The completed questionnaires were managed and stored securely at the Thai Binh University of Medicine and Pharmacy, Vietnam.

### Data collection

Data were collected using a structured and pilot-tested questionnaire. The questionnaire was in Vietnamese. The questionnaire was pretested with ICGs (N = 25) and validated to ensure the understandability and cultural relevance of the questions. Two village health workers from each health station in the 8 selected communes were trained as interviewers at a 2-day workshop followed by field-based training and counseling to administer the questionnaires (for a total of 16 interviewers). The interviews were arranged with assistance from staff from the commune health stations.

### Variables

#### Outcome variable

Burden related to caregiving when supporting a person with T2DM was based on the Zarit Burden Interview (ZBI)-8 questionnaire, which was developed by Arai et al [23]. The included questions were: 1) feeling stressed between caring for the person with T2DM and trying to meet other responsibilities for your family or work; 2) feeling strained when around the person with T2DM; 3) feeling uncertain about what to do about the person with T2DM; 4) feeling of not having enough time for oneself because of time spent with the person with T2DM; 5) worried and afraid of what the future holds for the person with T2DM; 6) not having enough money to take care of the person with T2DM; 7) feeling that one should be doing more for the person with T2DM; and 8) overall burden experienced by the person when caring for a person with T2DM.

ZBI-8 question numbers 1–7 were answered by applying a Likert scale with five answering options: 0 (never); 1 (rarely); 2 (sometimes); 3 (quite frequently); and 4 (nearly always). For ZBI-8 question number 8, the following answering options were used: 0 (not at all); 1 (a little); 2 (moderately); 3 (quite a bit); 4 (extremely). Total scores for the 8 burden-related items can range from 0 to 32 [24]. A higher total score indicates that the caregiver feels a higher burden.

#### Exposure variables

To assess factors and determinants associated with caregiver burden, we included variables, that in other studies have shown to be associated with caregiver burden: age, sex (male/female), relationship to the person living with diabetes (spouse/offspring/in-law/others) [25]; educational level (completed secondary school or less/high school/college or higher) [26] occupation (unemployed/farmer/employed/retired); monthly income [15]; residing with the person with T2DM (yes/no) [27]; duration of caring for the person with T2DM [14, 15]; whether the ICG was diagnosed with any chronic disease (yes/no); living with the person with T2DM (yes/no); and whether the ICG provided care to another person besides the person with T2DM (yes/no).

### Statistical analysis

Data were double entered using Epidata version 3.1 (the EpiData Association), cleaned and exported to SPSS (IBM Statistical Package for Social Science software) version 22 for analysis. Descriptive statistics were used to characterize the ICGs’ characteristics, and Spearman correlation and unadjusted linear regression analyses were performed to investigate the relationship between each characteristic. Quantile regression analyses were performed and included all characteristics that were found to be significant in the unadjusted model. The regression analysis for caregiver burden was performed for the 25th percentile (25%Q), median (50%Q) and 75th percentile (75%Q). The coefficient (coeff) value was shown in the regression table results, indicating the direction of the relationship between a predictor variable and the response variable. A positive coefficient indicates that when the predictor variable increases, the response variable also increases; a negative coefficient indicates that the response variable decreases when the predictor variable increases. All decisions on the statistical significance of the findings were made using *p* values ≤ 0.05 and 95% confidence interval (CI).

## Results

The general characteristics of ICGs are presented in Table 1. Among 1,241 ICGs, 51.1% were women. The median age of ICGs was 46.0 years. 27.3% of ICGs had completed high school, 46.3% had completed secondary school and 54.6% were employed. Of those, 611 (49.2%) had taken care of the person with T2DM for 5 years or longer and 855 (68.9%) lived in the same house as the person with T2DM. Of the ICGs, 44.3% were adult children of the person with T2DM (Table 1).

**Table 1:** Characteristics of caregivers and informal caregiver burden in groups (n = 1241).

Characteristic	Number of individuals (% of total) or median (p25/p75)	Caregiver burden score (median)	p
**Caregiver burden median (p25/p75))**	**7.3 (2.2/10.1)**		
**Age (Median)**	46 (37/61)		0.845*
**Monthly Income (Median)**	$173.9 (86.9/217.9)		<0.001*
**Sex**			0.528
Male	607 (48.9)	7.0	
Female	634 (51.1)	7.0	
**Education**			<0.001
Secondary school or less	575 (46.3)	8.0	
High school	338 (27.3)	8.0	
College or higher	328 (26.4)	3.0	
**Occupation**			<0.001
Unemployed	118 (9.5)	8.0	
Employed	678 (54.6)	7.0	
Farmer	311 (25.1)	9.0	
Retired	134 (10.8)	1.5	
**Relationship with person with T2DM**			0.370
Spouse	484 (39.0)	7.0	
Daughter/son	550 (44.3)	7.0	
Daughter-in-law/son-in-law	118 (9.5)	8.0	
Other	89 (7.2)	8.0	
**Duration of providing care to person with T2DM**			0.242
<5 years	581 (46.8)	8.0	
5–10 years	420 (33.8)	7.0	
>10 years	191 (15.4)	8.0	
Do not remember	49 (3.9)		
**Living in the same household as person with T2DM**			0.222
Yes	855 (68.9)	7.0	
No	386 (31.1)	7.0	
**Having a chronic disease**			0.001
Yes	650 (52.4)	7.5	
No	591 (47.6)	7.0	
**Engaged in care of at least one other person besides the person with T2DM**			<0.001
Yes	589 (47.5)	8.0	
No	652 (52.5)	6.0	

p25/p75: 25^th^ percentile/75^th^ percentile

*Spearman correlation

Median test was used for comparison in the groups

The minimum and maximum ZBI score range: 0–25

The results of the Spearman analysis and comparison of median in subgroups are presented in Table 1. ICGs with a college education or a higher educational status presented with lower scores of burdens as compared to ICGs who had completed high school or secondary school or less (8.0 versus 3.0) (p<0.001). Compared to retired and employed ICGs, farmers and unemployed ICGs presented with higher scores of burdens (p<0.001). ICGs who had a chronic disease themselves had higher burden scores as compared to those who had no chronic disease (7.5 vs 7.0) (p = 0.001). ICGs engaged in care of other persons besides the person with T2DM also presented with higher burden scores as compared to caregivers without disease and having no responsibility to support other persons (8.0 versus 6.0) (p<0,001). In addition, monthly income was negatively associated with burden of ICGs (p<0.001). Interestingly, providing care to a person with T2DM for five years or more was not associated with higher burden scores.

**Fig 2:**
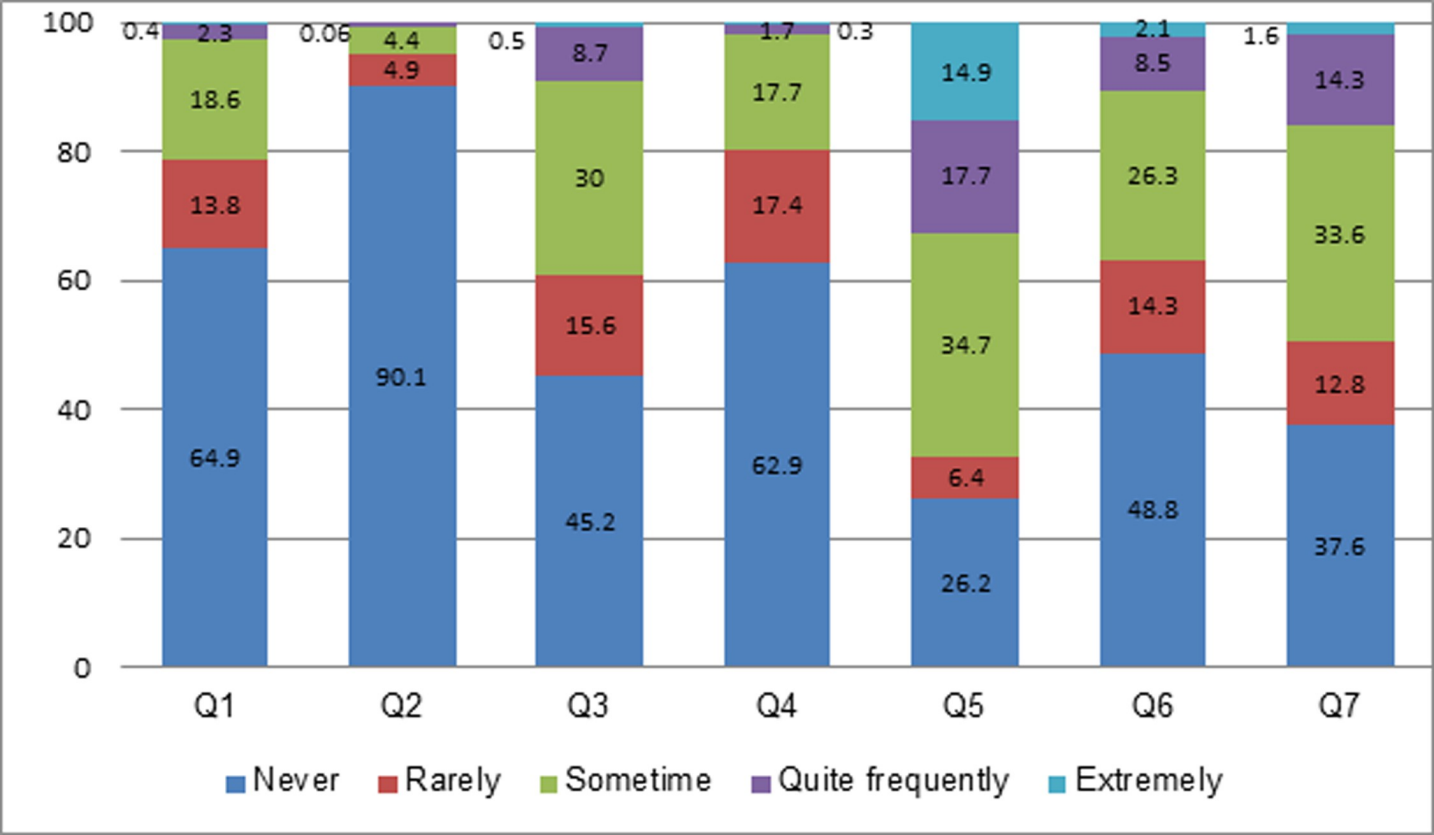
Responses to 7 burden questions (percentage). Q1: Do you feel stressed between caring for the person with T2DM and trying to meet other responsibilities for your family or work? Q2: Do you feel strained when around person with T2DM? Q3: Do you feel uncertain about what to do about person with T2DM? Q4: Do you feel that because of the time you spend with person with T2DM you do not have enough time for yourself? Q5: Are you afraid of what the future holds for the person with T2DM? Q6: Do you feel that you do not have enough money to take care of person with T2DM in addition to the rest of your expenses? Q7: Do you feel that one should be doing more for person with T2DM?

The sum of scores of caregiver burden ranged from 0 to 25 with a median score of 7.3 (25^th^ Percentile: 2.2; 75^th^ Percentile: 10.1). Of 1,241 participants, only 2.7% reported that they feel stress/torn quite frequently/nearly always between caring for persons with T2DM and meeting responsibilities for their family and work, 2.0% reported that they quite frequently/nearly always did not have sufficient time for themselves because of caring for the person with T2DM (Fig 2).

**Fig 3:**
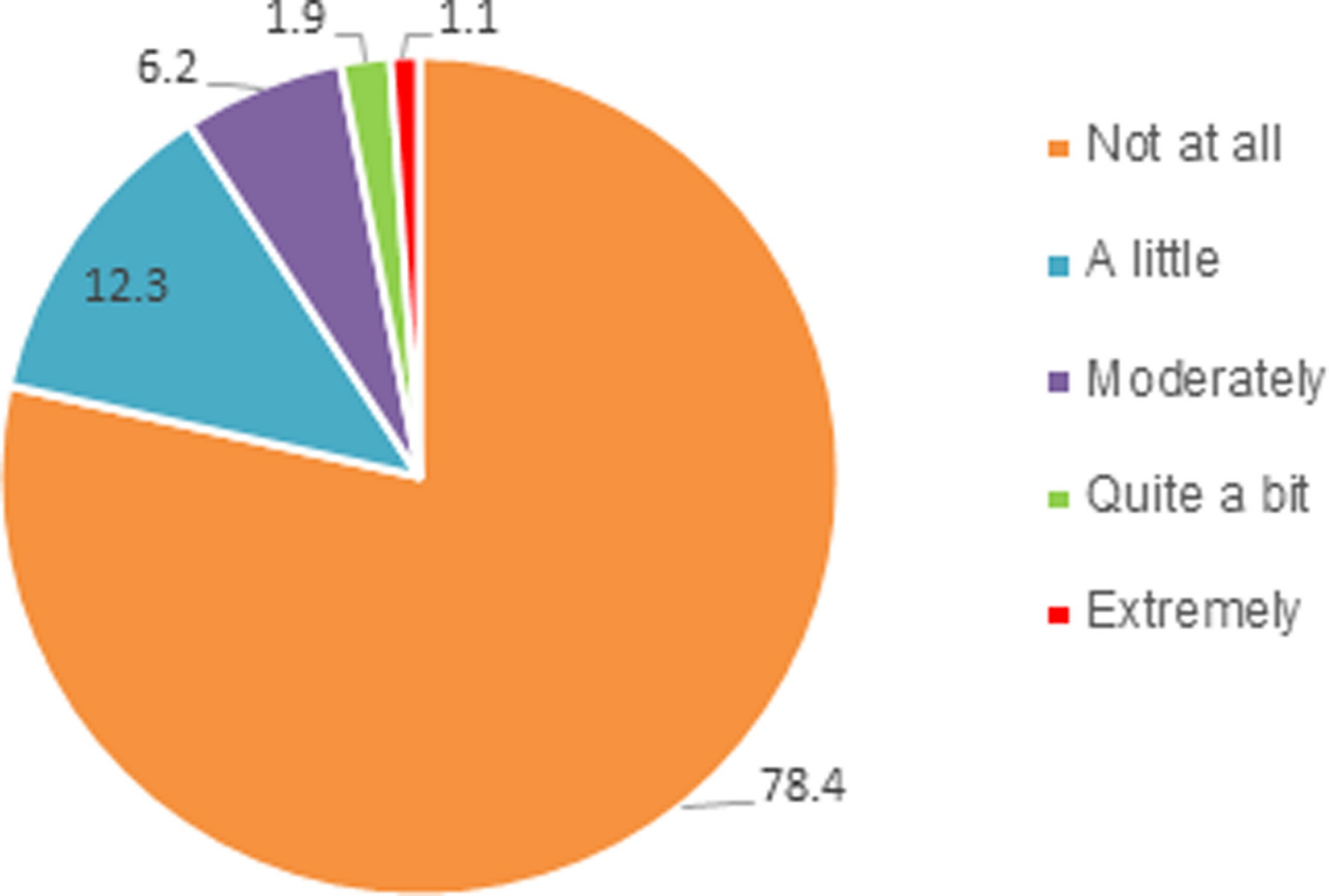
Responses to 8^th^ burden question (percentages). Q8) Overall, how often do you feel burdened by caring for person with diabetes?

In relation to the total burden (Q8), 6.2%, 1.9% and 1.1% reported that they felt moderately burdened, quite a bit burdened and extremely burdened due to their caregiving, respectively, 78% did not feel any burden at all (Fig 3).

In the adjusted quantile regression model, there was no association between monthly income and burden score in quantile 25, but in quantile 50 and quantile 75, the burden decreased by 0.4 points when income increased by $100. Compared with ICGs who graduated from college, ICGs who graduated from secondary school had burden scores that were 4.0, 3.0 and 2.2 points higher in the 25%Q, 50%Q and 75%Q, respectively (p<0.001). ICGs who provided care for other persons in addition to the person with T2DM had 2.0 points higher burden at the 25%Q, 2.54 points at the 50%Q and 1.66 points at the 75%Q as compared to caregivers who supported only the person with T2DM (p<0.001). ICGs who were farmers had 2.0 points higher burden scores as compared to employed counterparts in the 25%Q (p<0,001). In contrast, retired caregivers had significantly lower burden scores as compared to employed caregivers in all quantiles (lower 2 points, 3.36 points and 2.08 points in 25%Q, 50%Q and 75%Q respectively) (Table 2).

**Table 2:** Quantile regression results on the factors affecting burden of caregivers.

Variable	25^th^ percentile		50^th^ percentile		75^th^ percentile		
**Median**	2.29		7.30		10.13		
	Coeff^#^	95% CI	Coeff	95% CI	Coeff	95% CI
**Income (USD)**	-0.0015	-0.003; 0.003	-0.004**	-0.007; -0.001	-0.004*	-0.007; -0.001
**Education**						
Secondary school or less	4.00***	2.92; 5.07	3.00***	2.15; 3.84	2.16***	1.09; 3.24
High school	2.00***	0.96; 3.03	2.45***	1.63; 3.27	1.91	0.88; 2.95
College or higher	Ref^##^		Ref		Ref	
**Engaged in care of at least one other person besides the person with T2DM**						
No	Ref		Ref		Ref	
Yes	2.00***	1.25; 2.74	2.54***	1.96; 3.13	1.66***	0.92; 2.40
**Have chronic disease**						
No	Ref		Ref		Ref	
Yes	-0,048	-0.89; 0.89	0.54	-0.15; 1.25	0.58	-0.31; 1.47
**Occupation**						
Unemployed	1.0	-0.42; 2.42	-0.63	-1.75; 0.48	0.75	-0.67; 1.58
Famer	2.00***	0.91; 3.08	0.36	-0.48; 1.21	0.50	-0.58; 1.58
Retired	-2.00**	-3.37; -0.62	-3.36***	-4.44; -2.28	-2.08*	-3.45; -0.71
Employed	Ref		Ref		Ref	

^#^ Coefficient (coeff): shows how much the mean of the dependent variable changes given a one-unit shift in the independent variable while holding other variables in the model constant

^##^Ref: Reference

*p<0.05

**p<0.01

***p<0.001

## Discussion

To assess the burden of informal care among people supporting a person living with T2DM and potential socio-demographic predictors for this burden, we analyzed data from 1,241 ICGs in a rural population in Vietnam.

Regarding the burden of caregiving, we found that the mean ZBI score among ICGs was 6.9 (SD = 5.1), with a median of 7.3. This indicates that the burden score was not high, especially considering that the maximum burden could reach 32, by definition. The factors associated with caregiver’s burden was educational level, occupation, monthly income, being engaged in care of another person other than the person with T2DM and having a chronic disease, but not longer duration of care.

The relatively low average ZBI score recorded in this study may be explained by the limited prevalence of individuals with T2DM experiencing severe complications associated with their diabetes. In Vietnam, persons with T2DM who develop more severe complications are usually transferred from district hospitals to higher-level hospitals for treatment. Consequently, they are not represented in the sample included in the present study. Moreover, most persons with T2DM in Vietnam are elderly [28]. Previous studies conducted in rural areas of Vietnam have shown that more than 90% of elderly persons reported not needing assistance from others [29]. Furthermore, they often contribute by helping their children take care of the grandchildren [30]. A qualitative study among persons with T2DM in Vietnam also indicated that those living with diabetes would like their children to live happy, carefree lives, unburdened by the person’s diabetes and potential disability, thus many tried to be discreet about their disease and downplayed it in the presence of relatives [31]. These factors may explain the relatively low level of ZBI scores observed in the present study. In addition, Vietnamese culture has been influenced by Confucian philosophy in which relationships within the family are anchored in the concept of filial piety, which shapes parent’s expectations of old-age support from children [32]. This is manifested in devotion, close contact between parents and children, financial support and instrumental care, especially for aging and frail family members [32, 33]. Despite facing many challenges and feeling that the caring task is hard, caregivers in our study did not describe caring as a burden perhaps because caregivers regard caring for their loved ones as their responsibility, or perhaps because they hesitated to voice complaints [17].

Results of quantile analysis showed an association between burden of caregiving and educational status, occupation, monthly income and being engaged in care of another person besides the person with T2DM. However, it is important to emphasize that it is not known whether the observed associations reflect clinically significant differences. Therefore, we cannot conclude whether these observed variations in the burden of caregiving between groups have a meaningful impact on the caregivers.

Diabetes may comprise a substantial economic burden on persons with T2DM and their family members. Persons with T2DM can lose their ability to work but still need to pay for medication, dietary supplements, special food and health care services for diabetes [34]. In 2017, the annual cost per T2DM patient in Vietnam was estimated at US $264.10 [28]. When persons with T2DM have no pension and are financially dependent on their loved ones, the cost of diabetes treatment is a burden for their relatives since the average monthly income in rural Vietnam is fairly low, around US $100 per month [35]. This may explain why low-income ICGs presented with a higher burden of caring for T2DM persons as compared to those with higher levels of monthly income. These results are in line with the study by Sadik et al. among caregivers of diabetic foot persons with T2D in Turkey [15].

In the present study, ZBI scores were higher among ICGs with low educational status as compared to ICGs who had completed higher education. Several prior studies reported that burden of caregiving decreases with the increasing level of ICGs’ education [26, 36]. In general, higher educational levels have been associated with increasing levels of health knowledge [37]. Caregivers with a high level of education are also presumed to be more resourceful and problem-solving in caregiving others [38]. Most likely, it is a challenge for ICGs with lower education to access diabetes management information, for instance, appropriate diet recommendations, medication, handling of hypoglycemia and prevention of diabetes-related complications [16].

In the present study, the burden of caregiving among farmers was higher than that of employed caregivers, while retired caregivers reported a lower burden of caregiving as compared to those who were employed. This may be the result of lower incomes among farmers and lower levels of education as compared to employed and retired people. In 2018, a Vietnamese farmer had an annual income of USD 1,450 while the average Vietnamese person had an income of USD 2,200 [39]. The financial stress and lack of knowledge around treatment of diabetes may lead to increased burden of caregiving among farmers. The results of the present study indicated that ICGs who had to support individuals other than persons with T2DM had higher levels of burden than participants who had to take care of only a person with T2DM. Within the culture in rural Vietnam, with many generations living under one roof, adult children are responsible for taking care of their parents, parents-in-law and grandparents in addition to their children [33]. This potentially leads to physical and mental burnout among ICGs who provide care for several persons living with chronic diseases. A study from Malaysia found no association between caregiving burden and provision of care to other people [38]. However, the study in Malaysia was conducted in a hospital setting, and the ICGs were focused on taking care of a patient admitted to the hospital, whereas the present study was carried out in the community where caregivers have a responsibility to care for others such as their spouse, children or grandchildren in addition to caring for the patient.

### Study limitations

The current study has some limitations. Firstly, due to the cross-sectional design of our study, it was not possible to determine the causal association between exposure variables and burden of caregiving. Secondly, only caregivers of people who had received treatment for T2DM at district hospitals were included in the study, and thereby ICGs of persons who received diabetes treatment in regional or provincial hospitals were excluded. The latter group may present with more severe types of T2DM and co-morbidities, which demand a different level of caregiving, and this therefore may result in selection bias. Thirdly, research performed in areas with the highest diabetes rates may lead to bias. It is possible that persons with T2DM in these areas will receive more health communication, have better knowledge about the disease and therefore do not need as much care from the caregiver as persons with T2DM in other areas. Fourthly, the ZBI-22 questionnaire, while having been assessed as highly reliable and culturally adapted for the Vietnamese population, lacks a reliability evaluation for the short form we utilized. Although a pilot test was conducted before the research, this constitutes a limitation in the study. Lastly, this study was conducted in a rural area of Thai Binh Province of Vietnam and it is not known whether the results can be generalized to other populations.

## Conclusion

Burden scores were found to be higher among informal ICGs with lower educational levels, farmers, persons with low monthly incomes, and those caring for persons other than individuals with T2DM. In other words, caregivers who were already burdened by additional life stressors exhibited elevated burden levels. These findings underscore the importance of health care policies and programs that specifically address the needs and situations of caregivers facing resource constraints or high stress levels. The insights gained from this study may inform the development of interventions aimed at supporting ICGs in rural areas in Vietnam and other low and middle-income countries.

## Supporting information

S1 Data Data of study. (XLSX)

S1 Checklist STROBE checklist. (DOCX)
